# ADAM17 mediates OSCC development in an orthotopic murine model

**DOI:** 10.1186/1476-4598-13-24

**Published:** 2014-02-05

**Authors:** Fernando Moreira Simabuco, Rebeca Kawahara, Sami Yokoo, Daniela C Granato, Lucas Miguel, Michelle Agostini, Annelize ZB Aragão, Romênia R Domingues, Isadora L Flores, Carolina CS Macedo, Ricardo Della Coletta, Edgard Graner, Adriana Franco Paes Leme

**Affiliations:** 1 Laboratório de Espectrometria de Massas, Laboratório Nacional de Biociências, LNBio, CNPEM, Campinas 13083-970, Brazil; 2 Faculdade de Ciências Aplicadas, Universidade Estadual de Campinas, UNICAMP, Limeira, Brazil; 3 Faculdade de Odontologia de Piracicaba, Universidade Estadual de Campinas, UNICAMP, Piracicaba, Brazil; 4 Faculdade de Odontologia, Universidade Federal do Rio de Janeiro, UFRJ, Rio de Janeiro, Brazil

## Abstract

**Background:**

ADAM17 is one of the main sheddases of the cells and it is responsible for the cleavage and the release of ectodomains of important signaling molecules, such as EGFR ligands. Despite the known crosstalk between ADAM17 and EGFR, which has been considered a promising targeted therapy in oral squamous cell carcinoma (OSCC), the role of ADAM17 in OSCC development is not clear.

**Method:**

In this study the effect of overexpressing ADAM17 in cell migration, viability, adhesion and proliferation was comprehensively appraised *in vitro*. In addition, the tumor size, tumor proliferative activity, tumor collagenase activity and MS-based proteomics of tumor tissues have been evaluated by injecting tumorigenic squamous carcinoma cells (SCC-9) overexpressing ADAM17 in immunodeficient mice.

**Results:**

The proteomic analysis has effectively identified a total of 2,194 proteins in control and tumor tissues. Among these, 110 proteins have been down-regulated and 90 have been up-regulated in tumor tissues. Biological network analysis has uncovered that overexpression of ADAM17 regulates Erk pathway in OSCC and further indicates proteins regulated by the overexpression of ADAM17 in the respective pathway. These results are also supported by the evidences of higher viability, migration, adhesion and proliferation in SCC-9 or A431 cells *in vitro* along with the increase of tumor size and proliferative activity and higher tissue collagenase activity as an outcome of ADAM17 overexpression.

**Conclusion:**

These findings contribute to understand the role of ADAM17 in oral cancer development and as a potential therapeutic target in oral cancer. In addition, our study also provides the basis for the development of novel and refined OSCC-targeting approaches.

## Introduction

ADAM17 (*A D*isintegrin *A*nd *M*etalloproteinase) or TACE (TNF-alpha Converting Enzyme) is a surface membrane associated protein responsible for the cleavage of several membrane proteins, which is a biological cell process called shedding [1,2]. Among the shed proteins, ADAM17 releases ectodomains of important signaling molecules such as TNF-α, TGF-α, EGF, HB-EGF and VEGFR2 and adhesion molecules, such as L-selectin, syndecans, CAMs (cell adhesion molecules) and cadherins [1,3]. ADAM17 expression, likewise other ADAM family members, is up-regulated in many types of cancers, correlating with tumor progression and aggressiveness [4]. The molecules that are shed by ADAM17 are mostly signaling molecules that regulate cell proliferation, survival, migration and invasion properties associated with malignant cells resulted mainly from the crosstalk between ADAM17 and epidermal growth factor receptor (EGFR) [1,5]. Interestingly, EGFR is a widely studied oncogene in head and neck tumors [6] and agents targeting EGFR have emerged as a potential adjuvant therapy for OSCC [7,8]. Then, despite the close relationship between ADAM17 and EGFR, it is still not clear the role that ADAM17 plays in oral cancer development. Metalloproteinases are particular important in oral cancer progression, and squamous cell carcinoma of head and neck has been classified as the fifth most common type of cancer in the world [9].

To map the effect of ADAM17 overexpression in oral cancer, ADAM17-overexpressing cells have been subjected to *in vitro* analyses of proliferation, migration and adhesion and to orthotopic murine tumor formation, followed by MS-based proteomics and biological network analysis. Here we show that overexpression of ADAM17 in SCC-9 cells increases cellular viability, migration, adhesion and tissue collagenase activity. In addition, the ADAM17 knockdown decreased adhesion and proliferation in A431 cells. The SCC-9 cells have also been able to increase tumor size and proliferation in the orthotopic murine tumor model comparing to control (SCC-9 cells overexpressing GFP), and MS-based proteomics of those tumors revealed up-regulation of several Erk regulatory proteins, which are associated with the Erk phosphorylation. These results can open novel avenues for the understanding of the role of ADAM17 and its downstream signaling components in oral cancer development.

## Material and methods

### Cell lines

The human OSCC (oral squamous cell carcinoma) cell line, SCC-9, was obtained from American Type Culture Collection (ATCC), and cultured as recommended. SCC-9 cells are originated from a human squamous carcinoma of the tongue. Human Epidermoid Carcinoma A431 (epidermoid carcinoma cell line originated from skin) cell line was grown in Roswell Park Memorial Institute (RPMI) -1640 medium supplemented with 10% FBS and antibiotics at 37°C in a 5% CO_2_ air atmosphere.

### Generation of stably transfected cells

SCC-9 cells were stably selected for expression of ADAM17 (AD17) or GFP. Briefly, cells were transfected with pcDNA-ADAM-HA, kindly provided by Dr. Ulrich from Max-Planck Institute of Biochemistry [10], or control pcDNA-FLAG-GFP, using Lipofectamine PLUS (Invitrogen) following manufacturer’s instructions. After transfection, G418 antibiotic was added to cultures at a concentration of 0.8 mg/ml and incubated for about 2 weeks, until complete death of untransfected cells. Cells were then splitted and frozen as mix population stably transfected cells expressing ADAM17 or GFP.

### Generation of stably ADAM17 knockdown A431 cell line

The lentiviral particle production and transduction of A431 cells for ADAM17 knockdown were achieved with the Mission^®^ shRNA Vector pLKO.1-puro System using shRNA Plasmid pLKO.1-Neo-CMV-tGFP (Sigma-Aldrich). Experimental procedures were carried out according to the manufacturers’ instructions. After transduction, G418 antibiotic was added to cultures at a final concentration of 0.8 mg/ml and incubated for about one week, until complete death of untransfected cells.

### Orthotopic murine model of oral squamous cell carcinoma

SCC-9 cells stably expressing ADAM17 or GFP were grown until 75% confluence and 2.5 × 10^5^ cells in 20 μl of phosphate-buffered saline (PBS) were implanted into the right lateral portion of the tongue of 6- to 8-week-old male Balb/c nude mice (n = 3), using a syringe with a 30 gauge disposable needle (BD Biosciences). This procedure was approved by the Institutional Committee for Ethics in Animal Research of the University of Campinas. Mice were sacrificed 20 days after implantation and the tumor tissues either from SCC-9 cells overexpressing ADAM17 or GFP were immediately harvested.

### Tumors size measurement

Measurements of tumor size were made using a caliper and tumor volumes calculated as: volume = 0.5 × [major value] × [minor value]^2^[11]. Tumors were then frozen in dry ice for further analysis (n = 3).

### Proliferative activity of tumors by immunohistochemical expression of Ki-67

The proliferative activity of the orthotopic tumors was analyzed by immunohistochemical expression of Ki-67 using the monoclonal mouse anti-Ki67 (clone Mib1, Dako) diluted 1:200, followed by streptavidin-biotin peroxidase complex method (Biotinylated Link Universal Streptavidin-HRP, Dako). Positive cells were calculated by counting labeled nuclei (positive-cells) of six high power fields (magnification of 400×) from each case with the aid of the Image J software and expressing the data as percentage.

### Protein extraction from tumors and in solution trypsin digestion

Each tissue sample was homogenized with liquid nitrogen using mortar and pestle. Tissue protein from each tumor (n = 2) were separately resuspended in 100 μl of extraction buffer (8 M urea, 75 mM NaCl, 50 mM Tris, pH 8.2, protease inhibitor cocktail complete mini-EDTA free (Roche) and incubated at room temperature for 30 min under agitation. After centrifugation at 12,000 × g for 10 min at 4°C, the supernatant was quantified using the Bradford method [12].

Briefly, the extracted proteins were reduced (5 mM dithiothreitol, 25 min at 56°C), alkylated (14 mM iodoacetamide, 30 min at room temperature in the dark) and digested with trypsin (Promega). The peptides were purified on StageTips C18 [13], dried down in a vacuum concentrator and reconstituted in 0.1% formic acid.

### LC-MS/MS analysis and data analysis

An aliquot containing 2 μg of proteins was analyzed on a LTQ Orbitrap Velos mass spectrometer (Thermo Fisher Scientific) connected to nanoflow liquid chromatography (LC-MS/MS) by an EASY-nLC system (Proxeon Biosystem) through a Proxeon nanoelectrospray ion source as described by Aragão *et al.*, [14]. Briefly, the peptides were separated by a 2-90% acetonitrile gradient in 0.1% formic acid using an analytical column PicoFrit Column (20 cm × ID75 μm, 5 μm particle size, New Objective), at a flow of 300 nl/min over 212 min. All instrument methods for the LTQ Orbitrap Velos were set up in the data dependent acquisition mode. The full scan MS spectra (m/z 300–2000) were acquired in the Orbitrap analyzer after accumulation to a target value of 1e^6^. Resolution in the Orbitrap was set to *r* = 60,000 and the 20 most intense peptide ions with charge states ≥ 2 were sequentially isolated to a target value of 5,000 and fragmented in the linear ion trap by low-energy CID (collision induced dissociation) with normalized collision energy of 35%. Two independent biological samples of control tumor and tumor overexpressing ADAM17 were analyzed and they were run in duplicates.

The raw files were processed using the MaxQuant version 1.2.7.4 [15] and the MS/MS spectra were searched using the Andromeda search engine [16] against the Uniprot Human Protein Database (release July 11th, 2012; 69,711 entries) with a tolerance of 20 ppm for precursor ions and 1 Da for fragment ions were set parameters for protein identification and a maximum of 2 trypsin missed cleavage. Carbamidomethylation of cysteine was set as a fixed modification, and oxidation of methionine and protein N-terminal acetylation were chosen as variable modifications. Both peptide and protein identifications were filtered at a maximum of 1% false discovery rate. Bioinformatics analysis was performed using the software Perseus v.1.2.7.4 [15] available in the MaxQuant environment and reverse and contaminant entries were excluded from further analysis.

Protein abundance was calculated on the basis of the normalized spectral protein intensity (LFQ intensity). The data were converted into log2. Two independent experiments with two technical replicates for each condition were group in control tumor and tumor overexpressing ADAM17 and a paired t-test was applied for testing of differences in protein intensities between these groups.

### Biological network analysis

Differentially expressed proteins were uploaded into the Ingenuity Pathways (IPA; Ingenuity Systems) Knowledge Base as a tab-delimited text file of gene names. Biological networks were generated using their Knowledge Base for interactions between mapped Focus Genes (user’s list) and all other gene objects stored in the knowledge base. In addition, functional analysis of the networks was performed to identify the biological functions and/or diseases that were most significant to the genes in the network. The significance of functional enrichment was computed by a Fisher’s exact test (p < 0.05). A detailed description of IPA can be found on the Ingenuity Systems website.

### Immunoblotting

For identification of ADAM17 mature form, protein extraction was performed using detergent containing lysis buffer (50 mM Tris pH 7.4, 150 mM NaCl, 1 mM EDTA, 1% Triton X-100) supplemented or not with 20 μM BB-2516 and 10 mM 1,10-phenanthroline (Sigma), as described by McIlwain *et al.*, [17]. Cells were obtained from a confluent 10 cm plate and lysis was carried out on ice for 15 minutes followed by centrifugation at 12000 × g for 10 minutes. The supernatant was quantified and 40 μg of total protein was applied on SDS-PAGE.

For the expression analysis of EGFR and its phosphorylated form, the membrane protein enrichment was performed. Cells were lysed with syringe in non-detergent lysis buffer (25 mM Tris–HCl pH 7.5, 10 mM CaCl_2_) supplemented with protease and phosphatase inhibitors (1 mM PMSF, cocktail protease inhibitor, 10 mM sodium pyrophosphate, 1 mM beta-glycerophosphate, 1 mM Na_3_VO_4_, 1 mM NaF). The lysate was centrifuged for 200 × g for 10 min and the supernatant was submitted to ultracentrifugation under 100,000 × g for 1 h. The pellet was ressuspended in RIPA buffer (25 mM HEPES pH 7.5, 150 mM NaCl, 1 mM EDTA, 1% NP-40, 0.2% Sodium deoxycholate) containing phosphatase inhibitors.

After separation by SDS-PAGE, proteins were transferred onto nitrocellulose membrane (GE Healthcare). The nitrocellulose membrane was blocked with 5% BSA for 2 h and incubated with anti-HA (1:2,000; Sigma-Aldrich), anti-Erk (1:1,000; Santa Cruz Biotech), anti-phospho-Erk (1:1,000; Santa Cruz Biotech), anti-EGFR (1:1,000; Santa Cruz Biotech), anti-phospho-EGFR (1:1,000; Cell Signaling) antibodies for 2 h or overnight. The membranes were washed three times, each for 5 min, with 10 ml of Tris-buffered saline containing 0.1% Tween 20 and then reacted to horseradish peroxidase conjugated secondary antibodies (1:5,000) for 1 h. After three washes, the visualization of bands was achieved by chemiluminescence with the ECL kit (Amersham Biosciences).

### Real time quantitative PCR

Total RNA was obtained using the TRIzol reagent (Invitrogen) and 2 μg of total RNA were used for retrotranscription using the First-Strand cDNA Synthesis Kit (GE Healthcare). Real-time quantitative PCR for ADAM17 was performed using SYBR Green PCR Master Mix (Applied Biosystems), and the dissociation curves were performed to confirm the specificity of products. The threshold cycles (CT) values of target gene were normalized relative to glyceraldehydes-3-phosphate dehydrogenase gene, and relative expression ratios were calculated by the 2-ΔΔ Ct method. Three independent experiments were performed with triplicates. The following PCR primers were used: ADAM17 forward, 5′-GGACCCCTTCCCAAATAGCA-3′ and reverse 5′-ATGGTCCGTGAGATCCTCAAA-3′; GAPDH forward 5′-GAAGGTGAAGGTCGGAGTCAAC-3′ and reverse 5′- CAGAGTTAAAA GCAGCCCCTGGT-3′.

### Functional assays

#### ***Analysis of ADAM17 activity by HB-EGF shedding on AP reporter assay***

HB-EGF shedding assay was performed as described by Le Gall *et al.*, [18] with some modifications [19]. Briefly, HEK293 cells stably transfected with HB-EGF-AP were seeded into 100-mm dishes and co-transfected with transient pcDNA-ADAM17-HA or GFP vector (negative control). After 48 h, cells were trypsinised, counted to 3 × 10^5^ cells per well and seeded in 24 well-plate (Corning). In the following day, the cells were starved during 4 h and activated with PMA (50 ng/ml) during 30 min or 1 h in a phenol-free medium. The cleavage of HB-EGF-AP was measured after overnight incubation. Briefly, 100 μl of conditioned media were collected of each well and added to individual wells of a 96-well plate containing 100 μl of AP buffer (0.5 M Tris–HCl, pH 9.5, containing 5 mM p-nitrophenyl phosphate disodium, 1 mM diethanolamine, 50 μM MgCl_2_, 150 mM NaCl, 5 mM EDTA) and measured at 405 nm. Two independent experiments were performed with triplicates.

### Cell viability assay by MTT

SCC-9 GFP and SCC-9 ADAM17 cells were seeded in 96-well plates and incubated for 7 days. MTT (12 mM tetrazolium 3-(4, 5-dimethylthiazol-2)-2, 5-diphenyltetrazolium bromide) was added and cells were incubated for 4 h at 37°C, in the dark. The media were removed and 100 μl of HCl 1 N and isopropanol (1:25) was added in each well and incubated for 15 min at room temperature under gentle agitation. Finally, absorbance was measured at 595 nm. Three independent experiments were performed with triplicates.

### Transwell migration assay

SCC-9 GFP and SCC-9 ADAM17 cells were plated in the upper chambers of 8 mm pore transwells (HTS Transwell-96 Well Plate, Corning) after a starvation period of 4 h. The cells were allowed to migrate towards the lower chamber containing EGF at concentration of 100 ng/ml. At the end of the assay, cells at the top chamber were removed with a cotton swab and the cells at the bottom of the insert filter were fixed with 10% formaldehyde for 10 min, washed with PBS and stained with 1% toluidine blue solution in 1% borax for 5 min. The dye was eluted using 1% SDS and the absorbance was measured at 620 nm. Three independent experiments were performed with triplicates.

### Cell adhesion assay

SCC-9 GFP and SCC-9 ADAM17 cells or A431/untreated (mock), A431/control (scrambled) and A431/shRNA ADAM-17 were submitted to adhesion assay as described by Aragão *et al.*, [19]. Briefly, 10^6^ cells were plated in 100 mm dishes and another 96-well plate was coated with Matrigel™ (2 μg per well; BD Biosciences). After 24 h, cells were trypsinised and seeded in the coated 96-well plate, previously washed three times with PBS and blocked with 3% BSA (bovine serum albumin) during 2 h. The adhesion was evaluated during 1 h in serum-free media supplemented with 3% BSA, the wells were washed 3 times and cells were fixed with 10% formaldehyde. Cells were stained with 1% toluidine blue containing 1% borax for 5 min. The dye was eluted using 100 μl 1% SDS and the absorbance was measured at 620 nm. Three independent experiments were performed with five replicates.

### Bromodeoxyuridine-labeling (BrdU) index

A431/control (scrambled) and A431/shRNA ADAM-17 cells were plated in 96-well plates at a density of 10,000 cells per well in medium containing 10% of FBS. After 16 h, the cells were washed with PBS and cultured in serum-free medium for 24 h. Following serum starvation, the medium was replaced by medium containing 2% or 10% of FBS. Proliferation rates were determined 24 h after incubation by measuring BrdU incorporation into DNA, (Cell Proliferation ELISA BrdU Colorimetric, Roche Applied Science, Germany). Briefly, BrdU antigen was added to the cultures in 1:10 dilution and kept for 2 h at 37°C in 5% CO_2_. After incubation, the medium was removed and manufacturer’s protocol was followed. Absorbance was measured at 450 nm with correction at 690 nm. One experiment was performed with five replicates.

### Collagenase activity in tumors and conditioned media

Zymography was performed with total protein extract from tumors (3 μg) and A431 conditioned media (10 μg) scramble and knockdown for ADAM17. For A431 conditioned media, cells were washed three times in PBS and incubated for 24 h in the serum-free medium. The media were collected and the final concentration of 1 mM PMSF (phenylmethylsulfonyl fluoride) was added to the media. Briefly, cell debris were eliminated by centrifugation at 4,000 × g during 5 min at 4°C and subsequently concentrated using a 3,000 Da centrifugal filter (Millipore, Billerica, MA) at 4,000 × g at 4°C. Samples (proteins from tumor and conditioned media) were submitted to 1-D electrophoresis on 12% SDS-polyacrylamide gels containing 1 mg/ml gelatin under nonreducing conditions, and gelatinolytic activity was performed as previously described [20]. Gels were stained with Coomassie blue and destained. Gelatin digestion was identified as clear bands against a blue background. One experiment was performed for the analysis of tumor samples and two independent experiments were performed for the conditioned media.

### Statistical analysis

For the functional experiments, the Student’s *t*-test, Fisher’s exact test or ANOVA followed by Tukey test was used with the significance level stated at 0.05 (GraphPad Prism version 5 for Windows).

## Results

### SCC-9 cells overexpressing ADAM17 have higher sheddase activity on HB-EGF

**Figure 1:**
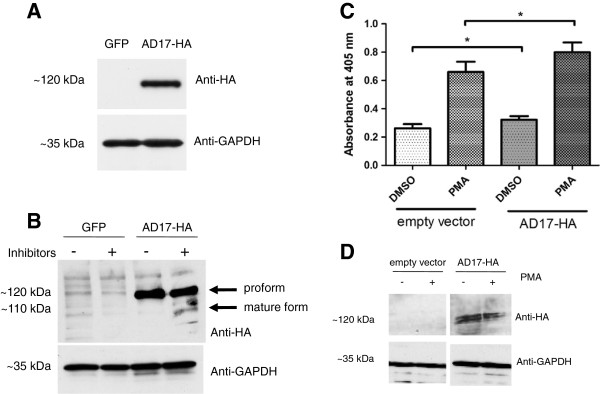
**ADAM17-HA (AD17-HA) has mature form in SCC-9 cells and increases shedding of HB-EGF in HEK293 cells. A**: SCC-9 cells were harvested, lysed with SDS sample buffer and immunoblotting was performed using anti-HA antibody. Anti-GAPDH antibody was used as loading control. **B**: SCC-9 cells were harvested and lysed in the presence and absence of BB-2516 and 1,10-phenanthroline. Immunoblotting was performed using anti-HA antibody and anti-GAPDH antibody was used as loading control. **C**: HEK293 cells overexpressing ADAM17-HA have increased shedding activity. HEK293 cells stably expressing a chimerical construct of HB-EGF fused with alkaline phosphatase (AP) were transfected with pcDNA-ADAM17-HA and released AP was quantified in cell supernatants after PMA (50 ng/ml) treatment. Two independent experiments with 3 replicates were performed (n = 2, Student’s *t*-test, DMSO: p = 0.0038, PMA: p = 0.0066). **D**: Immunoblotting of HEK293 cells overexpressing ADAM17-HA, performed using anti-HA antibody and anti-GAPDH antibody was used as loading control.

Stable overexpression of ADAM17-HA in SCC-9 cells was confirmed by immunoblotting (Figure 1A). SCC-9 cells overexpressing GFP (control) were selected in parallel and the expression was checked by fluorescence (data not shown). To show that overexpressed ADAM17-HA has its mature form, we performed cell lysis in the presence of BB-2516 and 1,10-phenanthroline. The result in Figure 1B indicates the expression of the mature form of ADAM17-HA in SCC-9 cells in the presence of those inhibitors.

In order to evaluate the activity of ADAM17-HA recombinant protein on cells overexpressing ADAM17, we have transfected pcDNA-ADAM17-HA in HEK293 cells stably expressing an HB-EGF-AP construct, which allows detection of shed HB-EGF in culture supernatants, a known target of ADAM17 [18]. The cells were stimulated by PMA and the results indicate increased shedding of HB-EGF (n = 2, Student’s *t*-test, DMSO: p = 0.0038, PMA: p = 0.0066) in cells transfected with empty vector or pcDNA-ADAM17-HA either stimulated or not with PMA. Immunoblotting performed as control indicates the same levels of ADAM17-HA and total proteins (Figure 1D).

### SCC-9 overexpressing ADAM17 shows higher cell viability, migration and adhesion

**Figure 2:**
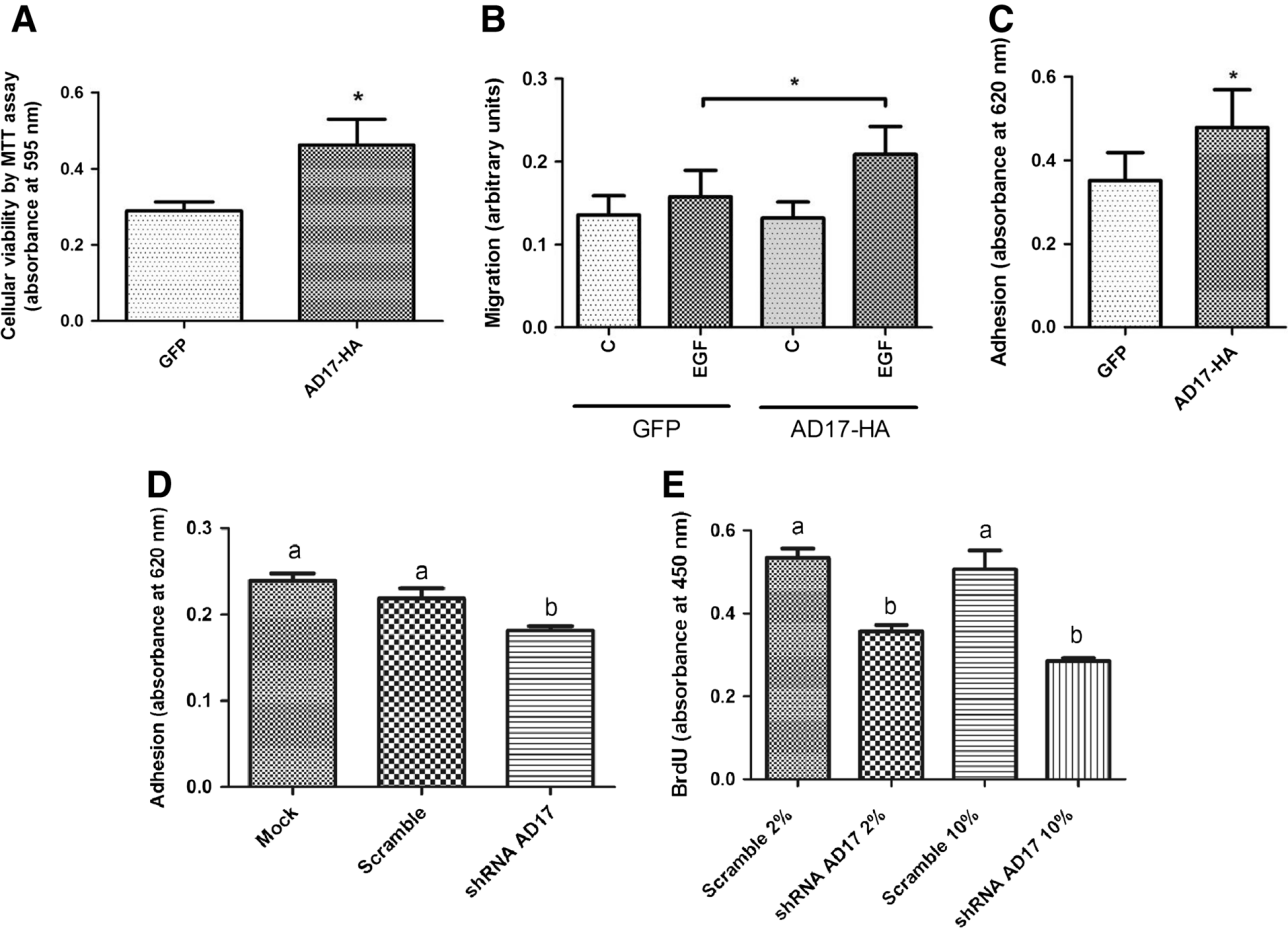
**ADAM17 regulates cellular viability, migration, adhesion and proliferation. A**: SCC-9 cells stably expressing ADAM17-HA or FLAG-GFP were seeded in 96-well plates. After 7 days cell viability was measured by MTT assay. Three independent experiments were performed (n = 3, Student’s *t*-test, p = 0.0004). **B**: SCC-9 cells stably expressing ADAM17-HA or FLAG-GFP were seeded in serum free media in the upper chamber of 96-well transwell plates. EGF at concentration of 100 ng/ml was added in serum free media in the lower chamber (n = 3, Student’s *t*-test, p = 0.0316). **C**: SCC-9 cells stably expressing ADAM17-HA or FLAG-GFP were seeded in Matrigel coated 96-well plates. After 1 h, cells were stained and adhesion measured (n = 3, Student’s *t*-test, p = 0.0001). **D**: ADAM-17 knockdown decreased adhesion of A431 cells. A431/untreated (mock), A431/control (scrambled) and A431/shRNA ADAM-17 cells were seeded in Matrigel coated 96-well plates. After 1 h, cells were stained and the cell adhesion was measured (n = 3, distinct letters represent significant differences at p < 0.0003, ANOVA followed by Tukey test). **E**: ADAM-17 knockdown decreased proliferation of A431 cells. Proliferation assay was performed in A431/control (scrambled) and A431/shRNA ADAM-17 cells by measuring BrdU incorporation into DNA in the presence of 2% or 10% FBS (n = 1, quintuplicate, distinct letters represent significant differences at p < 0.05, ANOVA followed by Tukey test).

SCC-9 cells overexpressing ADAM17-HA have been evaluated in viability, migration and adhesion assays. First, SCC-9 cells were seeded in 96-well plates and, after 7 days, cell viability was evaluated by MTT assay. SCC-9 cells overexpressing ADAM17-HA had increased viability compared with control (Figure 2A, n = 3, Student’s *t*-test, p = 0.0004).

For migration evaluation, SCC-9 cells overexpressing ADAM17-HA or GFP were seeded in 96-well transwell plates containing EGF in the lower chamber. After 24 h, migration to the lower chamber was measured by colorimetric assay. SCC-9 cells overexpressing ADAM17-HA showed an increased migration in the presence of EGF (Figure 2B, n = 3, Student’s *t*-test, p = 0.0316).

In adhesion assay, SCC-9 cells overexpressing ADAM17-HA or GFP were seeded in 96-well plats coated with Matrigel. After 1 h, cells were fixed, stained and adhesion was measured by colorimetric assay. As seen in Figure 2C, ADAM17-HA increased adhesion of SCC-9 cells (n = 3, Student’s *t*-test, p = 0.0001).

### ADAM17 knockdown promoted lower adhesion and proliferation in A431 cells

To further validate these data in another cell line, we have used A431 carcinoma cell line silenced for ADAM17 expression in adhesion assay. As shown in Figure 2D, knockdown of ADAM17 decreases adhesion of A431 cells (n = 3, p < 0.0003, ANOVA followed by Tukey test). We also performed a proliferation assay by measuring BrdU incorporation into DNA in the presence of 2% or 10% FBS and we observed lower proliferation in ADAM17 knockdown A431 cells compared with the control cells (Figure 2E, n = 1, quintuplicate, p < 0.05, ANOVA followed by Tukey test).

### Tumors overexpressing ADAM17 have increased size and showed higher proliferative activity

**Figure 3:**
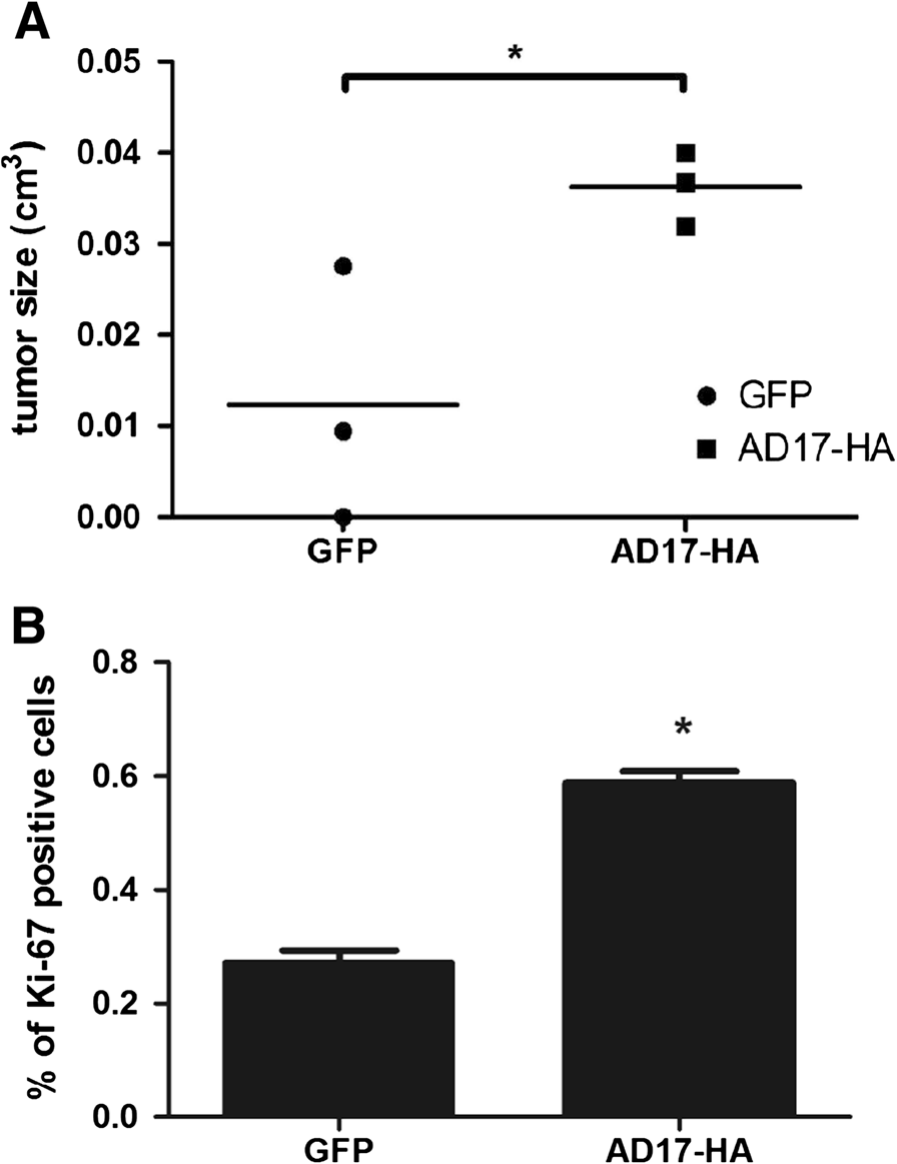
**SCC-9 cells overexpressing ADAM17-HA increased tumor size and proliferation. (A)** SCC-9 cells overexpressing ADAM17-HA (AD17-HA) induce increased size of tumors in Nude mice. Cells grown in tissue culture plates were trypsinized, resuspended in PBS and injected in the tongue of Nude mice. After 20 days, mice were sacrificed, tumors excised and measured (n = 3, Student’s *t*-test, p = 0.0467). **(B)** SCC-9 cells overexpressing ADAM17-HA (AD17-HA) induce higher proliferative activity by immunohistochemical expression of Ki-67. Positive cells were calculated by counting labeled nuclei (positive-cells) of six high power fields (magnification of 400×) from each case with the aid of the Image J software and expressing the data as percentage (n = 6, Student’s *t*-test, p < 0.0001).

An orthotopic murine tumor formation model using SCC-9 cells overexpressing ADAM17 or GFP was performed. After 20 days, tumors were excised and had their size measured. As seen in Figure 3A, tumors induced with SCC-9 cells overexpressing ADAM17 had increased size compared to SCC-9 GFP cells (n = 3, Student’s *t*-test, p = 0.0467). SCC-9 cells overexpressing ADAM17-HA (AD17-HA) induce higher proliferative activity by immunohistochemical expression of Ki-67 compared to SCC-9 GFP cells (n = 6, Student’s *t*-test, p < 0.0001) (Figure 3B).

### MS-based proteomics and biological network analysis indicate up-regulated proteins in the Erk pathway

**Figure 4:**
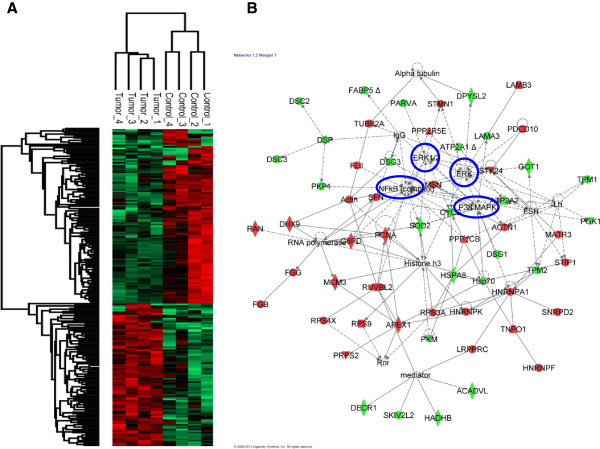
**Bioinformatic analysis of ADAM17-regulated proteome. (A)** Clustering of significantly up- and down-regulated proteins in tumor samples compared with control, Student’s *t*-test, p < 0.05, obtained in Perseus software. **(B)** Global interaction network by IPA consists of 56 (28%) of 200 differential expressed proteins, up-regulated proteins (red) and down-regulated proteins (green), plus additional interacting molecules that were not identified in this study (white). The two top biological networks generated by IPA were merged to obtain a global view. Major hubs in the network were highlighted in blue.

After protein extraction from tumors (n = 2) and trypsin digestion, mass spectrometry analysis was performed by LC-MS/MS, followed by protein identification using MaxQuant and analysis using Perseus software. A total of 2,194 proteins were identified at a false discovery rate (FDR) of less than 1%. The normalized spectral protein intensity (LFQ intensity) given by MaxQuant algorithm was converted into Log2 values. Normal distribution was verified by the histogram graph applied after normalization (Additional file 1: Figure S1). Correlation analysis between all of the individual replicates resulted in R values of at least 0.93, indicating high reproducibility among the samples (Additional file 1: Figure S2). We next performed statistical analysis to explore global proteomic difference between tumor overexpressing ADAM17 and control tumor samples. 200 proteins showed statistically significant expression (Student’s *t*-test, p < 0.05, Additional file 2: Tables S1 and Additional file 3: Table S2). Among them, 110 proteins were down-regulated and 90 were up-regulated in tumor tissues overexpressing ADAM17. Hierarchical clustering of significantly changing proteins was performed using the Z-score calculation on Log2 intensity values and it is represented as a heat map (Figure 4A).

To further explore the biological network of the identified proteins, we have examined functional pathway enrichment of the differentially expressed proteins by using Ingenuity Pathway Analysis (IPA) (Additional file 4: Table S3). 199 query molecules, out of 200, were eligible for network analysis based on the IPA Knowledge Base criteria. The top two networks have been merged to obtain a global view of the proteins that were differentially regulated between the tumor tissues: control and overexpressing ADAM-17 (Figure 2A). The global network contained 58 proteins from the input data out of 70. The network revealed protein interactions mainly in the context of cancer (p = 1.57E-06), solid tumor (p = 4.61E-06), carcinoma (p = 9.78E-06), proliferation (p = 2.00E-07) and cell death (p = 6.36E-05), encompassing more than 50% of the differential regulated proteins presented in the network, 39, 34, 33, 32, 28 respectively (Table 1). Additional file 4: Table S3 shows all functions and diseases related to the genes in the network and the respective p-value given by Fisher’s exact test. It is also interesting to obverse that Erk, Erk 1/2 and NF-κB and p38 MAPK represent the major hubs in the network, indicating some disrupted pathways by which the overexpression of ADAM-17 might be involved.

**Table 1:** **Top 10 functions and diseases annotation given by IPA, based on the number of proteins related to the network in Figure**4

**Function**	**Function annotation**	**p-value**	**Molecules**	**# Molecules**
Cancer	Cancer	1.57E-06	ACADVL, APEX1, ATP2A1, ATP2A2, CYCS, DHX9, DPYSL2, DSC3, DSG1, DSG3, DSP, FABP5, FGB, FGG, FLII, G6PD, GOT1, HNRNPA1, HNRNPK, HSPA8, LAMA3, LAMB3, LRPPRC, MCM3, PARVA, PCNA, PGK1, PKM, RAN, RPS4X, RPS9, SFN, SOD2, STIP1, STK24, STMN1, TPM1, TPM2, TUBB2A	39
Solid tumor	Solid tumor	4.61E-06	APEX1, ATP2A1, ATP2A2, DHX9, DPYSL2, DSC3, DSG1, DSG3, DSP, FABP5, FGB, FGG, FLII, G6PD, HNRNPA1, HNRNPK, HSPA8, LAMA3, LAMB3, LRPPRC, MCM3, PARVA, PCNA, PGK1, PKM, RPS9, SFN, SOD2, STIP1, STK24, STMN1, TPM1, TPM2, TUBB2A	34
Carcinoma	Carcinoma	9.78E-06	APEX1, ATP2A1, ATP2A2, DHX9, DPYSL2, DSC3, DSG1, DSG3, DSP, FABP5, FGB, FGG, FLII, G6PD, HNRNPA1, HNRNPK, HSPA8, LAMA3, LAMB3, LRPPRC, MCM3, PARVA, PCNA, PGK1, PKM, RPS9, SFN, SOD2, STIP1, STMN1, TPM1, TPM2, TUBB2A	33
Proliferation	Proliferation of cells	2.00E-07	ACTN1, APEX1, ATP2A2, DECR1, DSG3, DSP, FABP5, G6PD, HADHB, HNRNPA1, HNRNPF, HNRNPK, HSPA8, LAMA3, MCM3, PCNA, PDCD10, PGK1, PKM, PKP1, PRPS2, RAN, RPS3A, RPS4X, RPS9, SFN, SOD2, STK24, STMN1, TPM1, TPM2, TUBB2A	32
Cell death	Cell death	6.36E-05	APEX1, ATP2A1, ATP2A2, CYCS, DECR1, DHX9, DSG1, DSG3, DSP, G6PD, HNRNPA1, HNRNPK, HSPA8, LAMA3, MSN, PARVA, PCNA, PDCD10, PKM, RAN, RPS3A, RUVBL2, SFN, SOD2, STIP1, STK24, STMN1, TPM1	28
Apoptosis	Apoptosis	6.07E-06	APEX1, ATP2A1, CYCS, DECR1, DHX9, DSG1, DSG3, DSP, G6PD, HNRNPA1, HNRNPK, HSPA8, LAMA3, MSN, PARVA, PCNA, PDCD10, PKM, RPS3A, RUVBL2, SFN, SOD2, STIP1, STK24, STMN1, TPM1	26
Necrosis	Necrosis	1.96E-04	APEX1, ATP2A1, ATP2A2, CYCS, DECR1, DSG1, DSG3, DSP, G6PD, HNRNPA1, HNRNPK, HSPA8, LAMA3, MSN, PARVA, PCNA, PKM, RAN, RPS3A, SFN, SOD2, STMN1, TPM1	23
Adenocarcinoma	Adenocarcinoma	7.94E-03	ATP2A1, DHX9, DSC3, DSG1, DSG3, DSP, FGB, FLII, HNRNPA1, HNRNPK, HSPA8, LAMA3, LAMB3, LRPPRC, PCNA, PGK1, PKM, RPS9, SOD2, TUBB2A	20
Carcinoma	Carcinoma in lung	7.96E-03	APEX1, ATP2A1, DHX9, DSC3, DSG1, DSG3, DSP, FGB, FGG, HNRNPA1, HNRNPK, HSPA8, LAMA3, LRPPRC, PCNA, SOD2, STMN1, TUBB2A	18
Carcinoma	Carcinoma in lung	7.96E-03	APEX1, ATP2A1, DHX9, DSC3, DSG1, DSG3, DSP, FGB, FGG, HNRNPA1, HNRNPK, HSPA8, LAMA3, LRPPRC, PCNA, SOD2, STMN1, TUBB2A	18

### Erk activation was validated by immunoblotting in tumor tissue overexpressing ADAM17 and in A431 knockdown for ADAM17 gene

**Figure 5:**
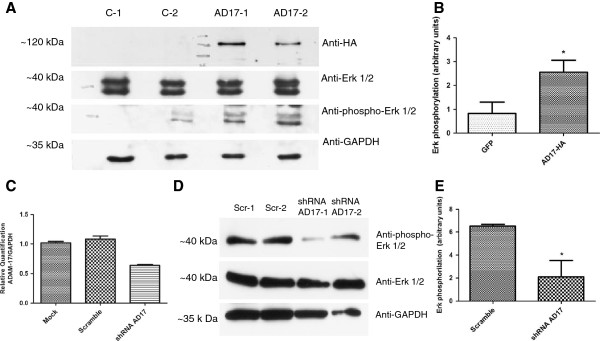
**Tumors induced by injection of SCC-9 cells overexpressing ADAM17 (AD17-HA) have increased Erk phosphorylation. A**: The immunoblotting indicates the expression of ADAM17-HA (anti-HA), total Erk (anti-Erk), phosphorylated Erk (anti-phospho Erk) and as loading control (anti-GAPDH). **B**: Phosphorylation levels were calculated by band intensity using ImageJ software (n = 2, Fisher’s exact test p = 0.0034). **C**: Knockdown of ADAM17 expression by shRNA in A431 cell line. Relative ADAM17 mRNA levels comparing shRNA and scrambled and mock control-treated cells were determined by quantitative RT-PCR. Each bar represents the mean ± SE of three independent experiments. **D**: shRNA-ADAM17 cells have shown a decreased in Erk phosphorylation. The immunoblotting indicates the expression of total Erk (anti-Erk), phosphorylated Erk (anti-phospho Erk) and as loading control (anti-GAPDH). **E**: Phosphorylation levels were calculated by band intensity using ImageJ software (n = 2, Fisher’s exact test p = 0.0001).

Immunoblotting has showed that Erk phosphorylation was increased in tumors overexpressing ADAM17 (Figure 5A-B, n = 2, Fisher’s exact test, p = 0.0034). To further validate these data, we used A431 carcinoma cell line knockdown for ADAM17 gene (Figure 5C) to analyze Erk phosphorylation state and it confirmed lower phosphorylation levels in Erk (Figure 5D-E, n = 2, Fisher’s exact test, p = 0.0001).

### SCC-9 cells overexpressing ADAM17 induced EGFR phosphorylation

**Figure 6:**
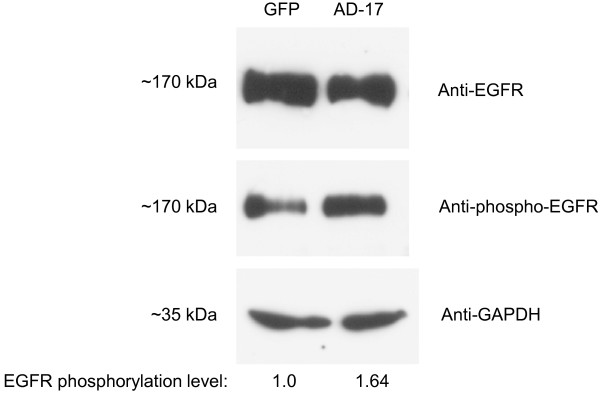
**EGFR shows increased activation in SCC-9 cells overexpressing ADAM17 (AD17-HA).** Immunoblotting showing increased EGFR phosphorylation in SCC-9 ADAM17-HA. Immunoblotting was performed using anti-EGFR, anti-phospho EGFR and anti-GAPDH antibodies. Phosphorylation levels were calculated by band intensity using ImageJ software and the GAPDH normalized intensity values are presented under the blots.

To investigate a downstream EGRF activation by SCC-9 cells overexpressing ADAM17, a total expression of EGFR and its phosphorylated levels have been evaluated. We first prepared enriched membrane protein extracts and then immunoblotting was performed. As shown in Figure 6, SCC-9 cells overexpressing ADAM17 have increased EGFR phosphorylation compared with SCC-9-GFP control cells.

### ADAM17 expression increases collagenase activity

**Figure 7:**
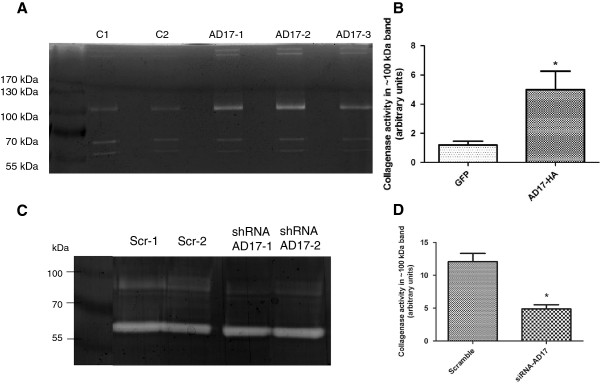
**Tumors induced by injection of SCC-9 cells overexpressing ADAM17 (AD17-HA) have increased collagenase activity.** C = tumors induced with SCC-9 overexpressing FLAG-GFP. ADAM17 = tumors induced with SCC-9 overexpressing ADAM17-HA. **A**: Collagenase activity is increased in SCC-9 overexpressing ADAM17-HA. **B**: Collagenase activity levels of ~100 kDa band in Figure **A** were calculated by band intensity using ImageJ software (n = 3, Student’s *t*-test p = 0.0285). **C**: shRNA-ADAM17 A431 cell conditioned media have reduced collagenase activity. **D**: Collagenase activity levels of ~100 kDa band in Figure **C** were calculated by band intensity using ImageJ software (n = 2, Student’s *t*-test p = 0.0370).

Collagenase activity of tumors has also been evaluated by zymography. After protein extraction from tumors, SDS-PAGE was performed in nonreducing conditions and it indicated higher collagenase activity for a ~100 kDa band in tumors overexpressing ADAM17 compared to control (Figure 7A and B, n = 3, Student’s *t*-test p = 0.0285). In addition, we have performed the analysis of collagenase activity in the secretome of A431 knockdown for ADAM17 and we confirmed the results, showing lower collagenase activity in the ~100 kDa compared to scramble shRNA cell line (Figure 7C-D, n = 3, Student’s *t*-test p = 0.0370).

## Discussion

In this report we have provided novel evidences demonstrating ADAM17 overexpression has a potential role in oral cancer development. To first address the concern of whether the overexpressed ADAM17-HA was active and functional, we validated the presence of mature form of the recombinant protein (Figure 1B), which is detected in the presence of inhibitors BB-2516 and 1,10-phenanthroline [17]. We were able to demonstrate an increase of HB-EGF shedding activity in cells overexpressing ADAM17 (Figure 1C). These results are in agreement with recent studies, which used these methods to demonstrate functionality and activity of ADAM17 [17,18].

In a second step, we examined the effect of ADAM17 overexpression in SCC-9 cells in events associated with oral cancer development. We indeed demonstrated that SCC-9 overexpressing ADAM17 showed an increase of cellular viability, migration and adhesion *in vitro* (Figure 2). Our data further demonstrated, by silencing ADAM17 expression, a decrease of adhesion (Figure 2D) and proliferation (Figure 2E) in A431 cells. These events dictated by ADAM17 were previously associated with other cancer cell lines [1,21-26].

ADAMs have been associated with many types of cancer, including brain, gastric, breast, prostate and lung [1]. Many models have been used to study ADAMs roles in cancer, for example, cells overexpressing ADAM12 were used in a tumor model in a previous study by Rocks *et al.*, [27], but it failed to induce tumors. In another study, knockdown of ADAM15 decreased malignant properties of prostate cancer PC-3 cells, such as migration and adhesion [21]. Although some studies have also shown an important role of ADAM17 in head and neck cancer [28-33], none of them investigated the ADAM17-mediated signaling components that might be involved in oral cancer development.

Firstly, we demonstrated, in an orthotopic tumor model for oral cancer, that tumors overexpressing ADAM17 presented an increase in size and showed higher proliferative activity by immunohistochemical expression of Ki-67 (Figure 3A and B, respectively). Secondly, to further provide the significance of ADAM17 in this process, MS-based proteomics were enabled to map some pathways regulated by ADAM17 in tumors induced by SCC-9 cells overexpressing this metalloproteinase. 2,194 proteins were identified in the tumors by MS and 200 proteins showed differential expression with p-value < 0.05. Amongst the regulated pathways found by biological network analysis, Erk signaling cascade was found predicted to be regulated (Figure 4). As expected, the Erk activation by phosphorylation was confirmed by immunoblotting (Figure 5). Erk is a key component of the MAP kinase cascade, which is triggered by growth factors and most of them are substrates of ADAM17 [1,3,22]. The signal transduction is mediated by a MAPK cascade, including Ras, Raf, MEK and the Erk 1/2 [34,35].

In addition, Erk pathway is a downstream signaling pathway of EGFR activation, transmitting several proliferative signals that bind and activate EGFR [5,34]. Some of the ligands of EGFR include important shed substrates of ADAM17, such as HB-EGF and TGF-α [1,3]. ADAM17 is a major sheddase responsible for EGFR signaling [1,5], which is a widely studied oncogene in head and neck tumors and an potential therapeutic target for OSCC treatment [7,8,36-38]. In fact, the overexpressing of ADAM17 in SCC-9 cells induced higher activation by phosphorylation of EGFR (Figure 6).

NF-κB pathway was also regulated by the overexpression of ADAM17 as shown in Figure 4B. NF-κB pathway is known to regulate the immune response to infection and it is also referred as a survival pathway of the cell, presenting a negative regulation of the apoptotic process [39]. Several reports show that dysregulation of NF-κB pathway is related to cancer, and recently to oral cancer development [40]. Other studies have also shown that different inhibitors of NF-κB pathway may be important to hinder tumor progression and to induce tumor cell death [41,42]. TNF-α, one of the main targets of ADAM-17, is known to initiate one of the signaling cascades that ultimately leads to NF-κB pathway activation, linking ADAM17 overexpression to NF-κB pathway regulation, as observed in our data [43]. Accordingly, our data provides additional support to the fact that NF-κB pathway is also involved in development of oral cancer.

The resulting effects of activation of EGFR, Erk and NF-κB pathways include regulation of cell adhesion, cell cycle progression, cell migration, cell survival, differentiation, metabolism, proliferation and apoptosis, which are all dysregulated in many cancer types [25,34,35,44-47]. These features were evidenced by the main function and disease annotations of the proteins associated with ADAM17 overexpression identified by MS (Table 1).

Further, we also observed that protein extracts from tumors overexpressing ADAM17 showed an increase of collagenase activity (Figure 7). Some studies had demonstrated that ADAM17 up-regulates other metalloproteinases, such as MMP-9 [48,49] and, interestingly, this activation was shown to be mediated by Erk pathway [50]. Despite a very complex signaling cascade, the increased collagenase activity found in the extracts of tumors overexpressing ADAM17 could also be associated to Erk phosphorylation, since Erk pathway was also activated in our model.

## Conclusion

In summary, our study shows that ADAM17 overexpression interferes in the biological processes associated with oral tumorigenesis and it is able to promote an increase tumor size and proliferation in an orthotopic murine model for oral cancer development. MS-based proteomics of tumors overexpressing ADAM17 indicated that the proteins modulated by ADAM17 are involved in the activation of Erk signaling pathway, which was also evidenced by EGFR activation and higher collagenase activity in tumors overexpressing ADAM17. Taken together, our findings indicate potential proteins regulated by ADAM17 overexpression and demonstrate the potential role of ADAM17 in the development of oral cancer. The understanding of the mechanism by which ADAM17 is associated with oral cancer progression will provide the basis for the development of novel and refined OSCC-targeting approaches.

## Competing interests

The authors declare that they have no competing interests.

## Authors’ contributions

Conceived and designed the experiments: FS, AFPL. Performed the experiments: FS, RK, SY, DG, LM, MA, AZBA, RR, ILF, CC, RDC. Analyzed the data: FS, RK, LM, RC, AFPL. Wrote the paper: FS, RK, RDC, EG, AFPL. All authors read and approved the final manuscript.

## Supplementary Material

Additional file 1: Figure S1 Normal distribution verified by the histogram of LFQ intensity-log2 transformed protein distribution in each sample. Figure S2. Correlation analysis between all of the individual replicates. The reproducibility of label-free quantification as illustrated by the LFQ intensity correlations. Click here for file

Additional file 2: Table S1 List of proteins identified by LC-MS/MS at a false discovery rate (FDR) of 1%. Protein abundance was calculated on the basis of the normalized spectral protein intensity (LFQ intensity) given by MaxQuant software. Click here for file

Additional file 3: Table S2 List of proteins identified by LC-MS/MS with LFQ intensity converted into Log2. Significantly expressed proteins given by Student’s *t*-test (p < 0.05) are shown by “+” label in *t*-test Significant column. Click here for file

Additional file 4: Table S3 List of functions and diseases given by IPA software in the genes showed in the network, p-value < 0.05. Click here for file

## References

[B1] Murphy G The ADAMs: signalling scissors in the tumour microenvironment Nat Rev Cancer 2008 8 929 941 19005493 10.1038/nrc2459

[B2] Gooz M ADAM-17: the enzyme that does it all Crit Rev Biochem Mol Biol 2010 45 146 169 10.3109/10409231003628015 20184396 PMC2841225

[B3] Edwards DR Handsley MM Pennington CJ The ADAM metalloproteinases Mol Aspects Med 2008 29 258 289 10.1016/j.mam.2008.08.001 18762209 PMC7112278

[B4] Duffy MJ McKiernan E O’Donovan N McGowan PM Role of ADAMs in cancer formation and progression Clin Cancer Res 2009 15 1140 1144 10.1158/1078-0432.CCR-08-1585 19228719

[B5] Blobel CP ADAMs: key components in EGFR signalling and development Nat Rev Mol Cell Biol 2005 6 32 43 10.1038/nrm1548 15688065

[B6] Sheu JJ-C Hua C-H Wan L Lin Y-J Lai M-T Tseng H-C Jinawath N Tsai M-H Chang N-W Lin C-F Lin C-C Hsieh L-J Wang T-L Shih I-M Tsai F-J Functional genomic analysis identified epidermal growth factor receptor activation as the most common genetic event in oral squamous cell carcinoma Cancer Res 2009 69 2568 2576 10.1158/0008-5472.CAN-08-3199 19276369

[B7] Bonner J Harari PM Giralt J Azarnia N Shin DM Cohen RB Jones CU Sur R Raben D Jassem J Ove R Kies MS Baselga J Youssoufian H Amellal N Rowinsky EK Ang KK Radiotherapy plus cetuximab for squamous-cell carcinoma of the head and neck N Engl J Med 2006 354 567 578 10.1056/NEJMoa053422 16467544

[B8] Huang S O Sullivan B Oral cancer: current role of radiotherapy and chemotherapy Med Oral Patol Oral y Cir Bucal 2013 18 e233 e240 10.4317/medoral.18772 PMC3613874 23385513

[B9] Petersen PE The world oral health report 2003: continuous improvement of oral health in the 21st century–the approach of the WHO global oral health programme Community Dent Oral Epidemiol 2003 31 Suppl 1 3 23 15015736 10.1046/j..2003.com122.x

[B10] Gschwind A Hart S Fischer O Ullrich A TACE cleavage of proamphiregulin regulates GPCR-induced proliferation and motility of cancer cells EMBO J 2003 22 2411 2421 10.1093/emboj/cdg231 12743035 PMC155989

[B11] Sobral LM Bufalino A Lopes M Graner E Salo T Coletta RD Myofibroblasts in the stroma of oral cancer promote tumorigenesis via secretion of activin A Oral Oncol 2011 47 840 846 10.1016/j.oraloncology.2011.06.011 21727023

[B12] Paes Leme AF Sherman NE Smalley DM Sizukusa LO Oliveira AK Menezes MC Fox JW Serrano SMT Hemorrhagic activity of HF3, a snake venom metalloproteinase: insights from the proteomic analysis of mouse skin and blood plasma J Proteome Res 2012 11 279 291 10.1021/pr2006439 21939285

[B13] Rappsilber J Mann M Ishihama Y Protocol for micro-purification, enrichment, pre-fractionation and storage of peptides for proteomics using StageTips Nat Protoc 2007 2 1896 1906 10.1038/nprot.2007.261 17703201

[B14] Aragão AZB Belloni M Simabuco FM Zanetti MR Yokoo S Domingues RR Kawahara R Pauletti B Gonçalves A Agostini M Graner E Coletta RD Fox JW Leme AFP Novel processed form of syndecan-1 shed from SCC-9 cells plays a role in cell migration PLoS One 2012 7 e43521 10.1371/journal.pone.0043521 22905270 PMC3419706

[B15] Cox J Mann M MaxQuant enables high peptide identification rates, individualized p.p.b.-range mass accuracies and proteome-wide protein quantification Nat Biotechnol 2008 26 1367 1372 10.1038/nbt.1511 19029910

[B16] Cox J Neuhauser N Michalski A Scheltema R Olsen JV Mann M Andromeda: a peptide search engine integrated into the MaxQuant environment J Proteome Res 2011 10 1794 1805 10.1021/pr101065j 21254760

[B17] McIlwain DR Lang PA Maretzky T Hamada K Ohishi K Maney SK Berger T Murthy A Duncan G Xu HC Lang KS Häussinger D Wakeham A Itie-Youten A Khokha R Ohashi PS Blobel CP Mak TW iRhom2 Regulation of TACE controls TNF-mediated protection against listeria and responses to LPS Science 2012 335 229 232 10.1126/science.1214448 22246778 PMC4250273

[B18] Le Gall SM Maretzky T Issuree PD Niu X-D Reiss K Saftig P Khokha R Lundell D Blobel CP ADAM17 is regulated by a rapid and reversible mechanism that controls access to its catalytic site J Cell Sci 2010 123 Pt 22 3913 3922 20980382 10.1242/jcs.069997 PMC2972273

[B19] Aragão AZB Nogueira MLC Granato DC Simabuco FM Honorato RV Hoffman Z Yokoo S Laurindo FRM Squina FM Zeri ACM Oliveira PSL Sherman NE Paes Leme AF Identification of novel interaction between ADAM17 (a disintegrin and metalloprotease 17) and thioredoxin-1 J Biol Chem 2012 287 43071 43082 10.1074/jbc.M112.364513 23105116 PMC3522302

[B20] Paes Leme AF Kitano ES Furtado MF Valente RH Camargo ACM Ho PL Fox JW Serrano SMT Analysis of the subproteomes of proteinases and heparin-binding toxins of eight bothrops venoms Proteomics 2009 9 733 745 10.1002/pmic.200800484 19137556

[B21] Najy AJ Day KC Day ML ADAM15 supports prostate cancer metastasis by modulating tumor cell-endothelial cell interaction Cancer Res 2008 68 1092 1099 10.1158/0008-5472.CAN-07-2432 18281484

[B22] Maretzky T Evers A Zhou W Swendeman SL Wong P-M Rafii S Reiss K Blobel CP Migration of growth factor-stimulated epithelial and endothelial cells depends on EGFR transactivation by ADAM17 Nat Commun 2011 2 229 21407195 10.1038/ncomms1232 PMC3074487

[B23] Zheng X Jiang F Katakowski M Lu Y Chopp M ADAM17 promotes glioma cell malignant phenotype Mol Carcinog 2012 51 150 164 10.1002/mc.20772 21480393 PMC3234333

[B24] Das S Czarnek M Bzowska M Mężyk-Kopeć R Stalińska K Wyroba B Sroka J Jucha J Deneka D Stokłosa P Ogonek J Swartz MA Madeja Z Bereta J ADAM17 silencing in mouse colon carcinoma cells: the effect on tumoricidal cytokines and angiogenesis PLoS One 2012 7 e50791 10.1371/journal.pone.0050791 23251384 PMC3519469

[B25] Lin P Sun X Feng T Zou H Jiang Y Liu Z Zhao D Yu X ADAM17 regulates prostate cancer cell proliferation through mediating cell cycle progression by EGFR/PI3K/AKT pathway Mol Cell Biochem 2012 359 235 243 10.1007/s11010-011-1018-8 21837402

[B26] Perna AF Sepe I Lanza D Capasso R Zappavigna S Capasso G Caraglia M Ingrosso D Hydrogen sulfide reduces cell adhesion and relevant inflammatory triggering by preventing ADAM17-dependent TNF-α activation J Cell Biochem 2013 114 1536 1548 10.1002/jcb.24495 23297114

[B27] Rocks N Estrella C Paulissen G Quesada-Calvo F Gilles C Guéders MM Crahay C Foidart J-M Gosset P Noel A Cataldo DD The metalloproteinase ADAM-12 regulates bronchial epithelial cell proliferation and apoptosis Cell Prolif 2008 41 988 1001 10.1111/j.1365-2184.2008.00557.x 19040574 PMC6496273

[B28] Ge L Baskic D Basse P Vujanovic L Unlu S Yoneyama T Vujanovic A Han J Bankovic D Szczepanski MJ Hunt JL Herberman RB Gollin SM Ferris RL Whiteside TL Myers EN Vujanovic NL Sheddase activity of tumor necrosis factor-alpha converting enzyme is increased and prognostically valuable in head and neck cancer Cancer Epidemiol Biomarkers Prev 2009 18 2913 2922 10.1158/1055-9965.EPI-08-0898 19843672 PMC2784191

[B29] Stokes A Joutsa J Ala-Aho R Pitchers M Pennington CJ Martin C Premachandra DJ Okada Y Peltonen J Grénman R James H Edwards DR Kähäri V-M Expression profiles and clinical correlations of degradome components in the tumor microenvironment of head and neck squamous cell carcinoma Clin Cancer Res 2010 16 2022 2035 10.1158/1078-0432.CCR-09-2525 20305301

[B30] Kornfeld J-W Meder S Wohlberg M Friedrich RE Rau T Riethdorf L Löning T Pantel K Riethdorf S Overexpression of TACE and TIMP3 mRNA in head and neck cancer: association with tumour development and progression Br J Cancer 2011 104 138 145 10.1038/sj.bjc.6606017 21102583 PMC3039790

[B31] Hinsley EE Hunt S Hunter KD Whawell S a Lambert DW Endothelin-1 stimulates motility of head and neck squamous carcinoma cells by promoting stromal-epithelial interactions Int J Cancer 2012 130 40 47 10.1002/ijc.25968 21491424

[B32] Yu C-C Tsai L-L Wang M-L Yu C-H Lo W-L Chang Y-C Chiou G-Y Chou M-Y Chiou S-H S-H miR145 Targets the SOX9/ADAM17 axis to inhibit tumor-initiating cells and IL-6-mediated paracrine effects in head and neck cancer Cancer Res 2013 71 3425 3440 23548270 10.1158/0008-5472.CAN-12-3840

[B33] M Andrade L Geraldo JM Nucleoplasmic calcium buffering sensitizes human squamous cell carcinoma to anticancer therapy J Cancer Sci Ther 2012 04 131 139

[B34] Steelman LS Franklin RA Abrams SL Chappell W Kempf CR Bäsecke J Stivala F Donia M Fagone P Nicoletti F Libra M Ruvolo P Ruvolo V Evangelisti C Martelli AM McCubrey JA Roles of the Ras/Raf/MEK/ERK pathway in leukemia therapy Leukemia 2011 25 1080 1094 10.1038/leu.2011.66 21494257

[B35] Roskoski R ERK1/2 MAP kinases: structure, function, and regulation Pharmacol Res 2012 66 105 143 10.1016/j.phrs.2012.04.005 22569528

[B36] Da Silva SD Ferlito A Takes RP Brakenhoff RH Valentin MD Woolgar JA Bradford CR Rodrigo JP Rinaldo A Hier MP Kowalski LP Advances and applications of oral cancer basic research Oral Oncol 2011 47 783 791 10.1016/j.oraloncology.2011.07.004 21802978

[B37] Wang D Su L Huang D Zhang H Shin DM Chen ZG Downregulation of E-Cadherin enhances proliferation of head and neck cancer through transcriptional regulation of EGFR Mol Cancer 2011 10 116 10.1186/1476-4598-10-116 21939503 PMC3192774

[B38] Guinea-Viniegra J Zenz R Scheuch H Jiménez M Bakiri L Petzelbauer P Wagner EF Differentiation-induced skin cancer suppression by FOS, p53, and TACE/ADAM17 J Clin Invest 2012 122 2898 2910 10.1172/JCI63103 22772468 PMC3408745

[B39] Baldwin AS Regulation of cell death and autophagy by IKK and NF-κB: critical mechanisms in immune function and cancer Immunol Rev 2012 246 327 345 10.1111/j.1600-065X.2012.01095.x 22435564

[B40] Page A Cascallana JL Casanova ML Navarro M Alameda JP Pérez P Bravo A Ramírez A IKKβ overexpression leads to pathologic lesions in stratified epithelia and exocrine glands and to tumoral transformation of oral epithelia Mol Cancer Res 2011 9 1329 1338 10.1158/1541-7786.MCR-11-0168 21821676

[B41] Lu K-W Tsai M-L Chen J-C Hsu S-C Hsia T-C Lin M-W Huang A-C Chang Y-H Ip S-W Lu H-F Chung J-G Gypenosides inhibited invasion and migration of human tongue cancer SCC4 cells through down-regulation of NFkappaB and matrix metalloproteinase-9 Anticancer Res 2008 28 1093 1099 18507059

[B42] Kwon H-K Hwang J-S So J-S Lee C-G Sahoo A Ryu J-H Jeon WK Ko BS Im C-R Lee SH Park ZY Im S-H Cinnamon extract induces tumor cell death through inhibition of NFkappaB and AP1 BMC Cancer 2010 10 392 10.1186/1471-2407-10-392 20653974 PMC2920880

[B43] Bouwmeester T Bauch A Ruffner H Angrand P-O Bergamini G Croughton K Cruciat C Eberhard D Gagneur J Ghidelli S Hopf C Huhse B Mangano R Michon A-M Schirle M Schlegl J Schwab M Stein MA Bauer A Casari G Drewes G Gavin A-C Jackson DB Joberty G Neubauer G Rick J Kuster B Superti-Furga G A physical and functional map of the human TNF-alpha/NF-kappa B signal transduction pathway Nat Cell Biol 2004 6 97 105 10.1038/ncb1086 14743216

[B44] Prenzel N Zwick E Daub H Leserer M Abraham R Wallasch C Ullrich A EGF receptor transactivation by G-protein-coupled receptors requires metalloproteinase cleavage of proHB-EGF Nature 1999 402 884 888 10622253 10.1038/47260

[B45] Zhang Q Thomas SM Xi S Smithgall TE Siegfried JM Kamens J Gooding WE Grandis JR SRC family kinases mediate epidermal growth factor receptor ligand cleavage, proliferation, and invasion of head and neck cancer cells Cancer Res 2004 64 6166 6173 10.1158/0008-5472.CAN-04-0504 15342401

[B46] McCubrey JA Steelman LS Chappell WH Abrams SL Wong EWT Chang F Lehmann B Terrian DM Milella M Tafuri A Stivala F Libra M Basecke J Evangelisti C Martelli AM Franklin RA Roles of the Raf/MEK/ERK pathway in cell growth, malignant transformation and drug resistance Biochim Biophys Acta 2007 1773 1263 1284 10.1016/j.bbamcr.2006.10.001 17126425 PMC2696318

[B47] Ma L Lan F Zheng Z Xie F Wang L Liu W Han J Zheng F Xie Y Huang Q Epidermal growth factor (EGF) and interleukin (IL)-1β synergistically promote ERK1/2-mediated invasive breast ductal cancer cell migration and invasion Mol Cancer 2012 11 79 10.1186/1476-4598-11-79 23083134 PMC3537707

[B48] Odenbach J Wang X Cooper S Chow FL Oka T Lopaschuk G Kassiri Z Fernandez-Patron C MMP-2 mediates angiotensin II-induced hypertension under the transcriptional control of MMP-7 and TACE Hypertension 2011 57 123 130 10.1161/HYPERTENSIONAHA.110.159525 21079048

[B49] Wang X Oka T Chow FL Cooper SB Odenbach J Lopaschuk GD Kassiri Z Fernandez-Patron C Tumor necrosis factor-alpha-converting enzyme is a key regulator of agonist-induced cardiac hypertrophy and fibrosis Hypertension 2009 54 575 582 10.1161/HYPERTENSIONAHA.108.127670 19581512

[B50] Xiao L-J Lin P Lin F Liu X Qin W Zou H-F Guo L Liu W Wang S-J Yu X-G ADAM17 targets MMP-2 and MMP-9 via EGFR-MEK-ERK pathway activation to promote prostate cancer cell invasion Int J Oncol 2012 40 1714 1724 22200661 10.3892/ijo.2011.1320

