# Supplementary figures and images for: ADAM17 mediates OSCC development in an orthotopic murine model
Source: Mol Cancer. 2014 Feb 5;13:24. doi: 10.1186/1476-4598-13-24 (PMC3928084; doi:10.1186/1476-4598-13-24)

## Slide 1
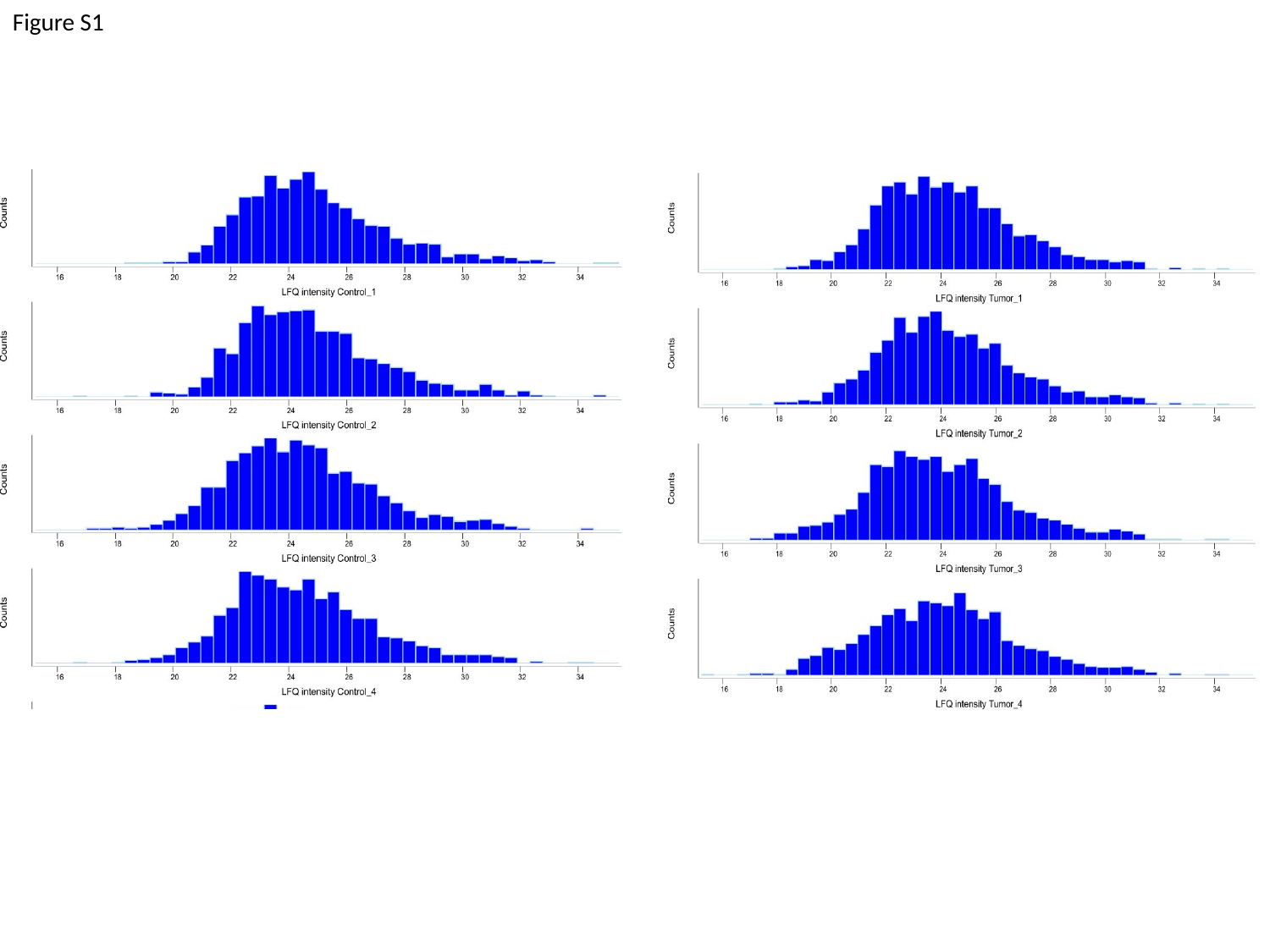

Figure S1

## Slide 2
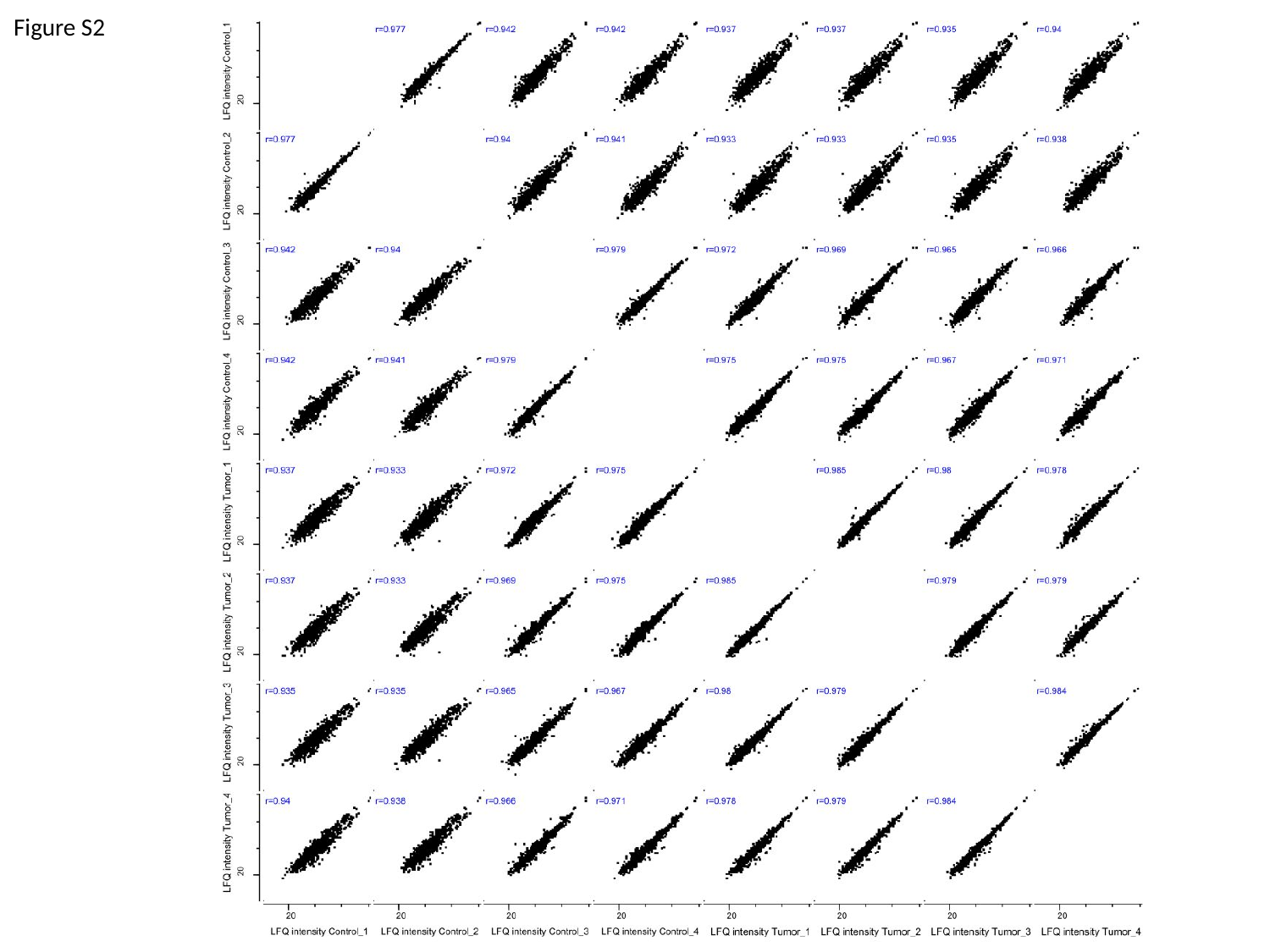

Figure S2

Supplement: Additional file 1: Figure S1 — Normal distribution verified by the histogram of LFQ intensity-log2 transformed protein distribution in each sample. Figure S2. Correlation analysis between all of the individual replicates. The reproducibility of label-free quantification as illustrated by the LFQ intensity correlations. [file 1476-4598-13-24-S1.ppt]
